# Supplementary material for: Key topics in pandemic health risk communication: A qualitative study of expert opinions and knowledge
Source: PLoS One. 2022 Sep 30;17(9):e0275316. doi: 10.1371/journal.pone.0275316 (PMC9524709; doi:10.1371/journal.pone.0275316)
Supplement: Attachment — Submitted filename: Response to reviewers 20.06.22.docx [file pone.0275316.s003.docx]

**Response to reviewers**

| Reviewer #1: This is a well written paper and I have only minor suggestions: 1. Please move duration of interviews to results section. | Thank you  Ok |
| --- | --- |
| 2. Please change 'UK variant' to 'alpha variant' | Corrected |
| 3. Please make the figures a bigger size so that they are readable. | The figures have been slightly increased in size. However, due to the amount of information included in the figures it is limited how much the bubbles can be enlarged. Therefore we included the animated version of the figures for readers to be able to view the bubble charts at larger size/on larger screen, please see S2 File ‘Key topics animated bubble chart’. |
| 4. Please consider shortening the results section. | We have shortened some of the results and the quotes and removed one quote. |
| Reviewer #2: The manuscript for this research article is well organized and clearly written. The overall study, construction, sample size, and design are very good.  The main claims are first, what exactly to communicate during a pandemic, in terms of risk, is highly related to the complexity, including scientific uncertainty, of communicating the message of a pandemic’s health risk. A second claim acknowledges integrating greater interdisciplinary expertise across expert domains between medicine/research and public health/communication that can offer possibilities for greater intelligibility of a situation at hand. Finally, a third recognizes mental models playing an important role in working through the first two points. All three are significant due to the ongoing issues surrounding pandemic health risk communication. Authors’ claims have been placed in the context of previous literature, albeit one article related to mental models is still under review (see reference 27). | Thank you for acknowledging our work |
| Please see specific comments below.  For clarity, the sentence on lines 50-52 should be rewritten, please note: • it has already been more than a century (1918-2022); • there have been at least four pandemic during that time period, including 1957-58 Pandemic Flu; • naming conventions of various pandemics are different, keep them consistent (e.g., use the disease name or the colloquial name, or utilizing both for each one), separately, if utilizing colloquial naming is undesirable across all diseases, consider extending this same care to nonhuman naming; and • consider renaming AIDS to HIV/AIDS. | This sentence has been rewritten to: ’Examples of pandemics include the Spanish Flu (1918–1919), HIV/AIDS pandemic (1966-), Swine Flu (2009–2010), and the COVID-19 pandemic (2019-).’ |
| Recommend utilizing “science expert” or “research expert” as one of the four inclusive categories throughout (see below for more detail). | Thank you for pointing out this inconsistency. We have corrected this throughout the paper. |
| • Table 1 presents five sample categories (1. medicine, 2. health research, 3. medical research, 4. public health, and 5. communication) that are then integrated into four categories in lines 224-225 (1. medicine, 2. medical/health researchers (science), 3. public health, and 4. communication). The second of these expert categories is then again combined, somewhat inconsistently, in various places in the manuscript, but especially lines 569-577 (i.e., “medical and science”, “medical/health”, and “medical and research”). | We have corrected this throughout the paper and we now only use the categories *medicine*, *public health* ,*science* and *communication experts*, which is consistent with figure 1. |
| Finally, if possible, but not absolutely necessary: • Briefly clarify/define, the author’s use of “interdisciplinary” (i.e., comparing and contrasting between disciplines) in this submission. | The use of “interdisciplinary” in this article refers to how it is necessary to work across the boundaries of different disciplines in pandemic health risk communication, and not merely rely on established disciplinary knowledge from different disciplines, aka in a “multidisciplinary” way.  As described under 4.1 Interdisciplinarity, the key topics in this article relate to concepts originating form different fields and disciplines, and we argue that a genuine interdisciplinarity is necessary to ensure coherence, clarity and engagement in communicating about these key concepts.  To exemplify the use of interdisciplinary approaches we added this sentence.  For example, to communicate a complex topic such as the R-value requires experts to work across the boundaries of different disciplines, e.g., statistics, medicine, pedagogy and marketing, which are all needed to together create and tailor the communication intervention. |
| • Note whether or not the levels of municipal, regional, and national offered any insights into how risks were perceived, contextualized, and/or communicated? (because, according to authors, there is no one-size-fits-all approach). | We have added the system levels (highlighted in yellow) to these following text in section 3.3.3:  The experts emphasised the significance of informing the public of the risk tradeoffs between primary and secondary consequences of the pandemic”. However, their perspectives on risk differed. The medical experts at hospital and regional level had low risk acceptance for the negative consequences of lockdown and isolation (e.g., increased mental illness among children and adolescents). Thus, they wanted all measures to be proportionate to the infection risk. Nevertheless, the public health expert in community emergency management had low risk acceptance for primary consequences of the pandemic, and thus communicated the importance of preventing the spread of the virus by implementing measures as early as possible:  We have also added the following text to section 4.2 in the discussion:  However, through interviewing participants at municipal, regional and national level, this study found that experts experienced different risk trade-offs related to primary and secondary consequences of the pandemic. Such risk tradeoffs may depend not only on their area of expertise, but also on their responsibility in the crisis management and their perspectives on risk and their proximity the to the hazard. Being transparent about such tradeoffs may introduce the risk of fragmented messages due to experts’ different perceptions of risk. When experts disagree, dissemination of risk tradeoffs may unintentionally compromise peoples’ trust by amplifying people’s perception of risk (15). Thus, communicating tradeoffs needs to be addressed differently depending on how each risk is perceived, contextualised and communicated by others in the crisis management system.  Finally, we have added the following text (highlighted in yellow) to section 4.3: There is no one-size-fits-all to the communication of health risk during a pandemic. There will always be unintended effects on some audience members and different considerations are taken at hospital, regional and national levels. |
