# Supplementary material for: Key topics in pandemic health risk communication: A qualitative study of expert opinions and knowledge
Source: PLoS One. 2022 Sep 30;17(9):e0275316. doi: 10.1371/journal.pone.0275316 (PMC9524709; doi:10.1371/journal.pone.0275316)
Supplement: Attachment — Submitted filename: Response to reviewers.docx [file pone.0275316.s004.docx]

Response to reviewers

| Reviewer #1: Thank-you for submitting your revisions.  With regards to the movement of interview length to the results section, I am not sure that where it is currently placed gives the best flow. I would suggest having a first section in the results summarising the respondent characteristics - table 1 and presenting the interview duration as part of this. | We agree that this information did not fit in where it was placed. We moved this information to the heading “interviews” in the method section. In qualitative articles it is normal to place information regarding sample characteristics and interview duration in the method section. |
| --- | --- |
| The animated figures as part of the supplementary material are a good idea. However, the figures in the main text cannot be viewed at the current size and so either they need to be simplified to allow bigger font or removed. | Thank you for notifying this issue. The Figures are enlarged and the text in Figures 1, 2 and 3 are now slightly enlarged. We choose to delete the main figure (previously named Figure 1), because the information is more readable in the zoomed-in figures. |
| Reviewer #2: Only minor updates necessary: • grammar: medical expert or expert in medicine, instead of "medicine expert" (e.g. line 281-282); | Corrected to expert in medicine |
| • category follow-up: change "medical/health research" to "medicine" (i.e., line 634); | Corrected to medicine |
| • extra "the" in line 576. | Corrected |
