# Supplementary material for: Key topics in pandemic health risk communication: A qualitative study of expert opinions and knowledge
Source: PLoS One. 2022 Sep 30;17(9):e0275316. doi: 10.1371/journal.pone.0275316 (PMC9524709; doi:10.1371/journal.pone.0275316)
Supplement: Attachment — Submitted filename: Response to reviewer.docx [file pone.0275316.s005.docx]

Response to reviewer

| Reviewer #1: Thank-you for your revisions. Two comments:  1. In medical journals it is standard practice to put the participant characteristics in the results section, even for qualitative papers. | We have moved participant characteristics to the result section. |
| --- | --- |
| 2. The text in the figures is still very small. | Images have been increased to 1920px wide, at 300dpi, and the text used in the images at full size is:  Font: Arial  Smallest text size: 22pt  Largest text size: 45pt  This adheres to the style guidelines for figures for PLOS one as stated in <https://journals.plos.org/plosone/s/figures> |
